# Discovering a Case of Lisinopril-Induced Necrotizing Pancreatitis

**DOI:** 10.7759/cureus.83239

**Published:** 2025-04-30

**Authors:** Anusha Majagi, Anshu Wadehra, Tooba Tariq, Bashar Mohamad

**Affiliations:** 1 Internal Medicine, Detroit Medical Center/Wayne State University, Detroit, USA; 2 Gastroenterology and Hepatology, Detroit Medical Center/Wayne State University, Detroit, USA

**Keywords:** acute necrotizing pancreatitis, acute pancreatitis complications, lisinopril-induced pancreatitis, medication-induced pancreatitis, side effects of lisinopril

## Abstract

Acute pancreatitis (AP) is one of the most common gastrointestinal diagnoses for inpatient admissions, with medications being a rare inciting agent. Our case highlights a 55-year-old male patient who was admitted for recurring epigastric pain and elevated lipase levels, with imaging showing AP with necrosis. After common etiologies for AP, including gallstones and alcohol, were ruled out, medication reconciliation showed that lisinopril was prescribed concurrently with the onset of AP episodes. Once lisinopril was discontinued, the patient's symptoms resolved. This case aims to make healthcare professionals aware of AP as a side effect of lisinopril.

## Introduction

Acute pancreatitis (AP) is one of the most common gastrointestinal diagnoses for inpatient admissions [1]. While alcohol and gallstones are the most common etiologies of AP, several medications can cause inflammation of the pancreas [2]. Drug-induced AP is a rare but important condition, making up less than 5% of all AP cases [2]. The most common medications resulting in drug-induced AP are azathioprine, sulfonamides, and diuretics such as furosemide and hydrochlorothiazide [3]. Pancreatic complications of AP include pseudocyst, a fluid sac forming outside of the pancreas, and pancreatic necrosis, which is tissue death of the organ [2]. Systemic complications include acute kidney injury and acute respiratory distress syndrome [2].

Lisinopril, an angiotensin-converting enzyme inhibitor (ACEi) that has been documented in the literature to induce pancreatitis, is a rare cause of AP, with only a handful of cases written. After an extensive literature review, there are only two other reports documenting necrotizing pancreatitis secondary to lisinopril use [2,4,5]. Our case demonstrates a patient with recurrent AP with evidence of pancreatic necrosis and pseudocyst formation in the setting of lisinopril use.

## Case presentation

A 55-year-old male with recurrent epigastric pain for one year presented to the hospital. Past medical history includes cholecystectomy, hypertension, type 2 diabetes mellitus, and peripheral artery disease. Home medications included lisinopril 10 mg daily, metformin 500 mg BID, cilostazol 100 mg daily, simvastatin 10 mg daily, and pantoprazole 40 mg twice daily. The patient reported no alcohol use. Familial history was negative for pancreatic malignancy. On admission, he was tachycardic at 115 beats per minute but otherwise hemodynamically stable. The physical exam findings included epigastric tenderness to palpation with muscle tension.

The positive workup included lipase, an enzyme to evaluate pancreatic activity, which was over 1000. Negative or normal laboratory investigations include the following: calcium, liver function tests (indicate biliary etiology), IgG4 (rule out autoimmune pancreatitis), and lipid profile (rule out hypertriglyceridemia); WBC, urinalysis, and chest X-ray (to rule out infection) were also obtained and within normal limits (Table 1). With epigastric pain and elevated lipase levels (1000+ U/L) on admission, we were able to diagnose our patient with AP.

**Table 1:** Pertinent laboratory data results for both admissions regarding AP ALT: alanine aminotransferase; AST: aspartate aminotransferase; WBC: white blood cells; IgG: immunoglobulin G; AP: acute pancreatitis

Lab Values	First Admission	Second Admission	Normal Values
Lipase	1,010	4,044	11-82 (U/L)
ALT	26	13	7-52 (U/L)
AST	21	18	13-39 (U/L)
Alkaline phosphatase	42	42	45-115 (U/L)
Bilirubin	0.48	0.48	<1.50 (mg/dL)
WBC	8.6	11	3.5-10.6 (K/mm^3^)
Glucose	119	84	75-105 (mg/dL)
Creatinine	1.05	0.99	0.70-1.30 (mg/dL)
Calcium	10.2	10.7	8.6-10.8 (mg/dL)
Triglycerides	106	-	<150 (mg/dL)
IgG subclass 4	37	-	1-291 (mg/dL)

**Figure 1:**
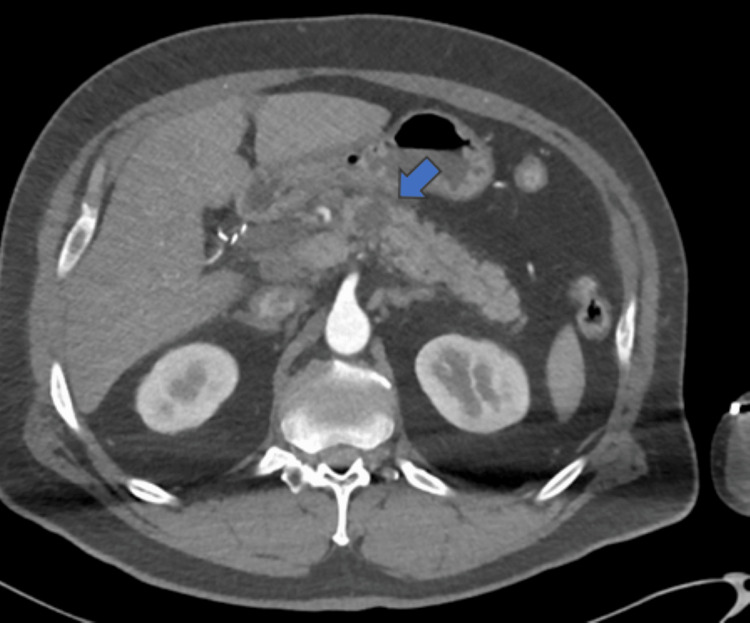
Computed tomography scan of the abdomen on first admission with a 1.8 cm hypoattenuating mass (blue arrow), identifying a possible pseudocyst, in the pancreatic body with related adjacent edema and fat stranding.

A CT abdomen/pelvis obtained on the first admission for pancreatitis showed a 1.8 cm round hypoattenuating structure in the pancreatic body, likely consistent with a pseudocyst (Figure 1). Additionally, the pancreatic head and uncinate process were edematous with adjacent fat stranding and reactive lymphadenopathy. No pancreatic ductal dilatation or gallstones were noted.

**Figure 2:**
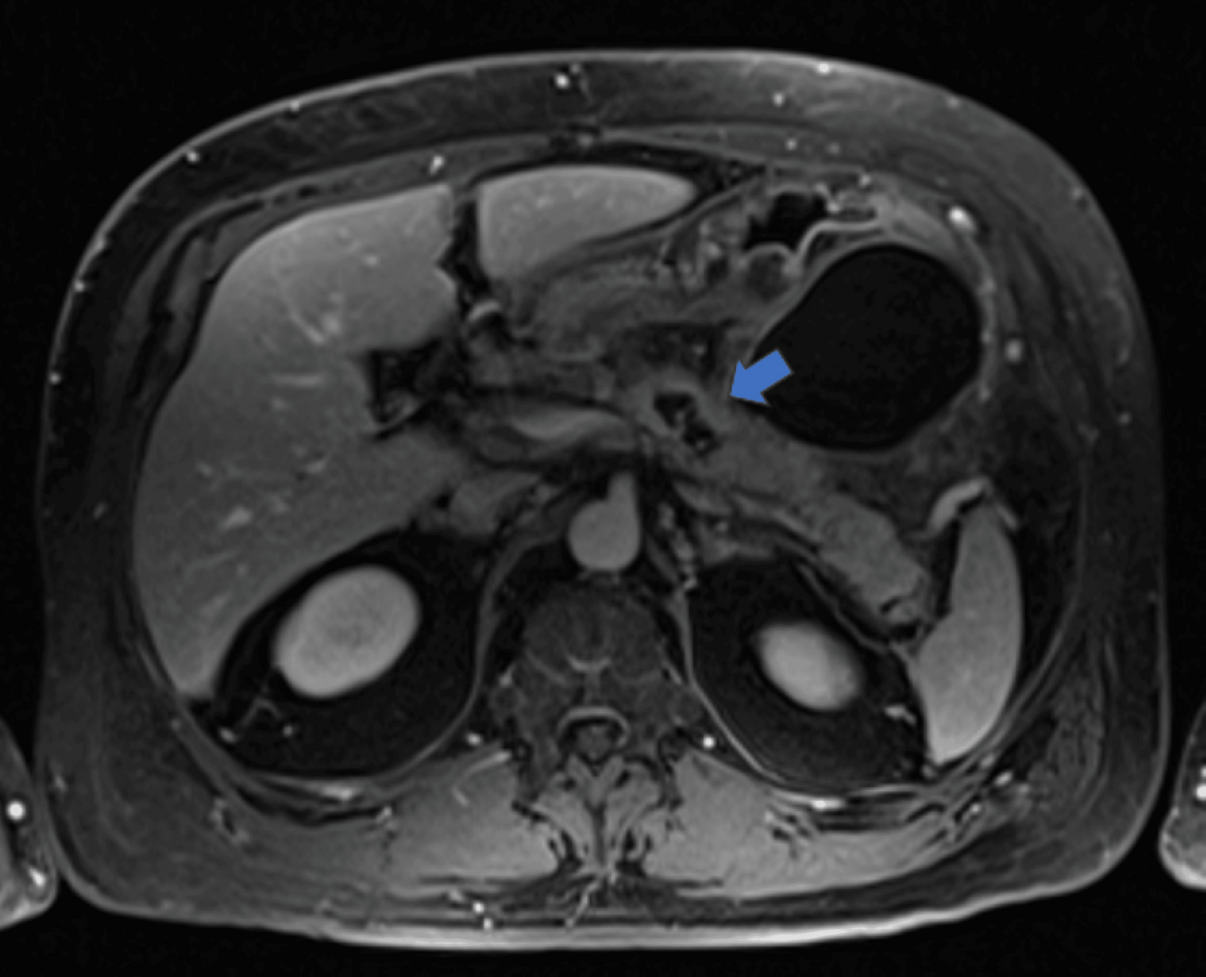
Magnetic resonance imaging-cholangiopancreatography obtained three weeks after Figure 1, with the blue arrow indicating necrosis of the pancreas.

The patient had a repeat admission for AP three weeks later. Aside from an increase in lipase levels and mild leukocytosis, there was no change in labs compared to the first admission (Table 1). MRI-cholangiopancreatography was obtained at the second admission for AP and showed acute necrotic pancreatitis (less than 30%) in the body of the pancreas with irregular extensions into the peripancreatic fat stranding, communicating with the main pancreatic duct (Figure 2). Biliary ducts were normal and without choledocholithiasis.

After a thorough electronic health record review and patient interview, it was discovered that the patient was prescribed lisinopril about one year ago, around the time of his symptom onset. Medication review did not reveal any other new medications started or medications commonly known to cause AP. Once lisinopril was discontinued, the patient's abdominal pain was greatly improved in subsequent outpatient follow-up visits, with no symptoms of AP for a one-year follow-up period. The patient was not re-challenged for ethical reasons and had no further episodes of AP.

## Discussion

Lisinopril is an ACEi that is medically indicated as a first-line treatment for hypertension [6]. It is also recommended to be used by the American Diabetes Association for hypertension in the setting of diabetes, given its reno-protective effect [7]. Other uses of this ACEi include improving survival in ST-segment elevation myocardial infarctions and adjuvant treatment for heart failure. Common side effects include angioedema, dry cough, and hyperkalemia, while it is also contraindicated in pregnancy given its teratogenic effects [6].

Drug-induced pancreatitis makes up roughly 0.1-2% of all AP cases, with lisinopril being an exceptionally rare inciting agent [3]. Consequently, it was imperative to rule out potential etiologies, such as gallstones, alcohol use, hypertriglyceridemia, hypercalcemia, traumatic inciting events, or autoimmune disorders, through comprehensive history-taking and lab work to make our final diagnosis.

While lisinopril-induced pancreatitis has been documented in case reports, only two other cases of lisinopril-induced necrotizing pancreatitis have been reported, indicating the rarity of this event [5,8]. In one case, the patient was taking lisinopril for eight months, with an unknown dose, before developing severe necrosis of the pancreas resulting in multiorgan failure and death [8]. The other case does not specify the duration of lisinopril use; however, the patient's dose was 20 mg, while our patient was taking 10 mg [5]. There may be a potential dose-dependent relationship between lisinopril dosing and pancreatitis, as seen in another case with pancreatitis complicated by pseudocyst after a two-fold increase in dosing 10 years after lisinopril was initiated [4]. Given the relatively low dose of lisinopril our patient was on, this may explain why it took about a year for symptoms to present.

While a definitive link between ACEi and AP has not been established, several possible mechanisms have been suggested [3]. One potential mechanism includes ACEi, which leads to the accumulation of bradykinin and increased vascular permeability, causing ductal obstruction [9]. As a result, pancreatic enzymes are confined within the pancreas, leading to autodigestion of pancreatic tissue. Further in the renin-angiotensin-aldosterone system, angiotensin II receptors are thought to play a role in circulation and secretion of pancreatic enzymes; with ACEi inhibiting angiotensin II receptor activation, AP symptoms could be aggravated [10]. Notably, a population-based study in 2017 found that there was a diminished risk of AP with angiotensin II receptor blockers (ARBs) [11]. ARBs should be considered over ACEi if the patient has a secondary indication for the latter, such as protective heart- and kidney-related benefits.

Our patient repeatedly presented with symptoms of epigastric pain, significant lipase elevation, and positive imaging findings for AP. Other common causes, including gallstones, heavy alcohol use, and metabolic abnormalities, were ruled out. The patient was not on any other medications that are known to cause AP. The Naranjo scale is a widely used tool to assess the likelihood of an adverse event being caused by a drug [12]. This patient presented with a Naranjo score of 8, indicating that these recurrent AP episodes were probably related to daily lisinopril use [12]. After the ACEi was discontinued, our patient's symptoms resolved, and he did not have further episodes of AP.

## Conclusions

Our case demonstrates how lisinopril can result in AP, even at normal therapeutic doses. When working up etiologies of AP, a thorough medication reconciliation should be performed to assess for potential offending medications. This case proves that, while rare, ACEi should be considered in the differential diagnosis when determining the etiology of AP in a patient if common causes are ruled out. Healthcare providers should be cognizant of and recognize AP as a possible side effect of lisinopril.
